# Long-term outcomes five years after selective dorsal rhizotomy

**DOI:** 10.1186/1471-2431-8-54

**Published:** 2008-12-14

**Authors:** Eva Nordmark, Annika Lundkvist Josenby, Jan Lagergren, Gert Andersson, Lars-Göran Strömblad, Lena Westbom

**Affiliations:** 1 Division of Physiotherapy, Department of Health Sciences, Lund University, Lund, Sweden; 2 Children's hospital, University Hospital, SE-221 85 Lund, Sweden; 3 Division of Paediatrics, Department of Clinical Sciences (Lund), Lund University, Lund, Sweden; 4 Division of Clinical Neurophysiology, Department of Clinical Sciences (Lund), Lund University, Lund, Sweden; 5 University Hospital, SE-221 85 Lund, Sweden; 6 Division of Neurosurgery, Department of Clinical Sciences (Lund), Lund University, Lund, Sweden

## Abstract

**Background:**

Selective dorsal rhizotomy (SDR) is a well accepted neurosurgical procedure performed for the relief of spasticity interfering with motor function in children with spastic cerebral palsy (CP). The goal is to improve function, but long-term outcome studies are rare. The aims of this study were to evaluate long-term functional outcomes, safety and side effects during five postoperative years in all children with diplegia undergoing SDR combined with physiotherapy.

**Methods:**

This study group consisted of 35 children, consecutively operated, with spastic diplegia, of which 26 were Gross Motor Function Classification System (GMFCS) levels III–V. Mean age was 4.5 years (range 2.5–6.6). They were all assessed by the same multidisciplinary team at pre- and at 6, 12, 18 months, 3 and 5 years postoperatively. Clinical and demographic data, complications and number of rootlets cut were prospectively registered. Deep tendon reflexes and muscle tone were examined, the latter graded with the modified Ashworth scale. Passive range of motion (PROM) was measured with a goniometer. Motor function was classified according to the GMFCS and measured with the Gross Motor Function Measure (GMFM-88) and derived into GMFM-66. Parent's opinions about the children's performance of skills and activities and the amount of caregiver assistance were measured with Pediatric Evaluation Disability Inventory (PEDI).

**Results:**

The mean proportion of rootlets cut in S2-L2 was 40%. Muscle tone was immediately reduced in adductors, hamstrings and dorsiflexors (p < 0.001) with no recurrence of spasticity over the 5 years. For GMFCS-subgroups I–II, III and IV–V significant improvements during the five years were seen in PROM for hip abduction, popliteal angle and ankle dorsiflexion (p = 0.001), capacity of gross motor function (GMFM) (p = 0.001), performance of functional skills and independence in self-care and mobility (PEDI) (p = 0.001).

**Conclusion:**

SDR is a safe and effective method for reducing spasticity permanently without major negative side effects. In combination with physiotherapy, in a group of carefully selected and systematically followed young children with spastic diplegia, it provides lasting functional benefits over a period of at least five years postoperatively.

## Background

Many children with cerebral palsy (CP) have spasticity that interferes with motor function and activities in daily life. A variety of treatment options to reduce spasticity have been developed [1,2]. As CP is a heterogeneous condition, the treatment should be highly individualized. Finding the right treatment at the right time for the child is challenging [3]. The only treatment option to reduce spasticity permanently is selective dorsal rhizotomy (SDR). This neurosurgical procedure can be used for the relief of spasticity interfering with motor function in children with CP. As the optimal goal of the operation is to improve function, it should always be combined with physiotherapy. SDR was introduced in North America in the early 1980s and the history of the procedure has been reviewed elsewhere [4-7]. The operation is usually recommended for young children with CP spastic diplegia, without dyskinesia or ataxia, without significant cognitive disability and with walking capacity or ambulation within reach [8-12]. SDR was introduced in Scandinavia in 1993 and is one of several treatment options at Lund University hospital for young and carefully selected children with spastic diplegia.

SDR combined with physiotherapy has been proven to be beneficial in the domains of body function and structure according to the International Classification of Functioning, Disability and Health (ICF) [13]. Lower limb spasticity is consistently relieved and lower limb range of motion increased [9,10,12,14,15]. Strength and gait speed are gained [16,17]. Evidence for positive impact in activities and participation has been reported [15,18-20]. Meta-analysis data derived from three randomized studies [9,10,12] concluded that SDR in combination with physiotherapy had a positive effect on gross motor function one year after the operation. The children who had undergone SDR and physiotherapy had a greater functional improvement than children only receiving physiotherapy. A direct relationship between percentage of dorsal rootlets transected and functional improvement was found [21]. Lasting functional improvements in gait have been shown 10 and 20 years after SDR [22,23].

SDR may reduce the need for orthopaedic procedures in patients with spastic CP if the SDR operation is conducted before the age of 5 years [24]. SDR has been suggested to have a positive, rather than negative, effect on the prognosis of hip subluxation [25]. With respect to the safety of SDR, perioperative complications are rare [26]. However, a high incidence of spinal deformities has been reported after SDR [27,28]. We are not aware of any previous long-term follow-up study after SDR with a complete consecutively operated group of children. The aims of this study were to evaluate long-term functional outcomes, safety and side effects during five years postoperative follow-up in a group of children undergoing SDR combined with physiotherapy who were consecutively operated and systematically monitored.

## Methods

### Patients

CP is defined as a motor-impairment syndrome secondary to early non-progressive lesions or anomalies of the brain [29]. The CP subtype spastic diplegia has more involvement of the legs than the arms, with or without walking ability within reach [30]. At the University Hospital of Lund, SDR is indicated in patients presenting with spasticity as the dominating neurological sign. All children (n = 35) with CP spastic diplegia undergoing SDR in the period March 1993 to June 1999 were included in the study. Preoperative gross motor function was classified, with the Gross Motor Function Classification System (GMFCS), into a five level ordinal scale where level I represent the least and level V the greatest functional limitations [31]. The mean age at operation was 4.5 years (range 2.5–6.6 years).

The medical team consisted of neurodevelopmental paediatricians (JL, LW) and physiotherapists (EN, ALJ) and the selection procedure included thorough and repeated preoperative clinical evaluations. No neurosurgeon was involved in the selection procedure. The child's motor activities and performance were assessed in many situations including provocation of different neurological signs at rest and in intensive activities. Selective motor control, alignment and muscle strength were assessed in provoked functional activities against gravity e.g. in different positions on a Bobath ball, repeated sit-to-stand and squatting. Neuroimaging was not performed to guide the selection for SDR during these years.

All children were monitored and examined by the same team pre- and at 6, 12, 18 months, 3 and 5 years postoperatively. The physiotherapist from the local child habilitation team and both parents participated in the selection assessments and discussions pre- and postoperatively.

Preoperative characteristics of the children are presented in Table 1. Perinatal asphyxia was defined as Apgar score ≤ 3 at 5 minutes and/or hypoxic ischemic encephalopathy with convulsions during the first 72 hours. Epilepsy was defined as active epilepsy requiring medical treatment. Information about the cognitive function was updated at the five year postoperative follow-up. Severe cognitive disability was defined as learning disability (mental retardation) corresponding to Intelligence Quotient (IQ) below 50, moderate cognitive disability to IQ between 50 and 70.

**Table 1:** Patients.

**Child characteristics**	**n = 35**
Boys/girls	24:11
Age at operation: mean, SD, range (years)	4.5, 1.1, (2.5–6.6)
Preoperative GMFCS levels:	
I	1
II	8
III	10
IV	15
V	1
Gestational age (completed weeks):	
< 26	3
26–27	2
28–31	19
32–36	4
37–42	5
> 42	0
Premature, gestational age not specified	2
Birth weight (g): mean, range	1689 (710–3370)
Twin/triplet	7/1
Perinatal asphyxia: yes/no/unknown	3/28/4
^a ^Shunted hydrocephalus: yes/no	8/27
^b ^Intraventricular haemorrhage: yes/no/not studied	10/8/17
^c ^Periventricular atrophy: yes/no/not studied	13/6/16
Any of ^a, b, c ^above: yes/no/not studied	21/6/8
Epilepsy pre-op: yes/no	5/30
Cognitive disability: severe/moderate/no or minor	3/7/25
Severe visual impairment	3
Overweight/underweight preoperatively	2/11
Overweight/underweight 5 years postoperatively	4/4

Severe visual disability was defined as functional blindness or binocular acuity of < 0.3 after correction of refraction errors.

The Swedish reference population growth curves were used [32]. Overweight was arbitrarily defined, in this study, as Body Mass Index (BMI) > + 2 SD for age and gender, underweight as BMI < -2 SD and/or weight for age and gender < – 2.5 SD.

### Selection criteria for SDR

Children below seven years of age with CP subtype spastic diplegia were selected for the operation. Spasticity interfering with present and future motor function and activities in daily life was considered an indication in all GMFCS levels, also when walking ability was out of reach. It was important that the child received and was willing to receive physiotherapy pre- and postoperatively. Cognitive disability was not considered a contraindication as long as the child had a drive to move and play. Contraindications were the presence of dystonia, ataxia, fixed contractures or earlier orthopaedic operations other than adductor tenotomy.

### Treatment goals

The family and the child's local therapist actively participated in the pre- and postoperative discussions and decisions regarding realistic expectations, goal setting and interventions. Individual functional goals for the intervention were set. The overall goals were to improve motor and functional skills e.g. improvement of alignment, balance in sitting and standing, transfers, walking and/or wheeled mobility. Additional desirable effects from the spasticity reduction were prevention of severe contractures, hip dislocation and pain.

For the children with independent walking ability GMFCS levels I–II the functional goals were to improve balance, endurance, flexibility in standing, walking, running jumping. For children in GMFCS level III goals were directed towards stability and variability in sitting, attain and maintain standing, walking and enabling self-propelled wheeled transfers. For the children in GMFCS levels IV–V the functional goals were independent sitting, supported standing and enabling of wheelchair transfers. Other goals, although not scientifically evaluated, were to reduce pain emerging from spasticity and inactivity as well as the daily care burden for the caregivers.

### Postoperative physiotherapy

After the operation, the child stayed in the intensive care unit for 3–5 days. Following transferal to the children's hospital, daily physiotherapy was started on the 5^th ^postoperative day and continued for a total of three weeks: one week at the Children's hospital and the two following weeks at the regional habilitation centre.

The first week of physiotherapy treatment focused on mobilization: rolling, sitting, functional activities and standing for periods of 45 minutes twice a day. During the second and third week of physiotherapy, a further session of 45 minutes was added daily. The physiotherapy was incorporated into the child's daily activities, promoting functional skills in playing, dressing, grooming, transfers and mobility. Hydrotherapy was introduced as soon as the scar had healed.

After discharge from the habilitation centre, the local therapist continued to implement functional activities and participation in daily activities. The recommended frequency of individualized treatment sessions for the first six months was one hour twice a week and thereafter once a week. In addition, physical leisure activities were encouraged. However, hippotherapy was not recommended until six months postoperatively.

Two months after discharge, the patient was seen by the neuropaediatrician and physiotherapist to support the rehabilitation process. All information collected during the pre-, peri- and postoperative assessments was documented in the medical records and study protocols.

### Neurosurgery and anesthesia

The children were premedicated with midazolam. General anaesthesia was induced with intravenous fentanyl and thiopental. Succinylcholine was given to facilitate tracheal intubation, followed by one dose of muscle relaxant. Anaesthesia was maintained with isoflurane/N2O/O2 and fentanyl. All children had a urinary bladder catheter. The operation technique described by Peacock was used [7,33,34]. A block laminoplasty was performed from L1 to L5. The dura was opened and the cauda equina exposed. The posterior roots were identified and the level was confirmed by visible anatomical features and by using electrical stimulation. Each root was divided into rootlets and stimulated. This process was facilitated by the invention of a new instrument, in the shape of a comb, where the rootlets could be placed and kept separated.

Postoperatively, the children were either treated with intrathecal (IT) morphine and rectal paracetamol every 6 hours or continuous infusion of IT bupivacain and morphine by a pump. All children received supplementary intravenous cetobemidon if necessary. Dose reduction was initiated 64 h after the operation and the IT treatment was terminated 48 h later as previously described [35]. All children received prophylactic intravenous antibiotics postoperatively.

### Neurophysiology

EMG recordings were obtained from the adductor, vastus lateralis, tibialis anterior, hamstrings and gastrocnemius muscles bilaterally, as well as from the external anal sphincter. Two silver wires, insulated except for the tip, were inserted into each muscle belly. In the external anal sphincter (EAS), they were inserted at 3 and 9 o'clock, and in the hamstrings, one wire was inserted into the medial and one into the lateral hamstrings. Stimulation of the roots was done through handheld cauterizing forceps connected to a constant current stimulator, pulse width 0.1 ms. The electromyography (EMG) responses were recorded with a Nihon-Kohden electroencephalogram recorder, paper speed of 20 mm/s. The amplifier had a low pass filter at 120 Hz and a time constant at 0.003s. Initially, ventral roots were stimulated with single pulses (usually 0.1–0.3 mA) to identify the S1 and S2 roots. In addition to EMG responses, the legs were inspected visually to detect toe flexion. Stimulation of the S2 root evoked responses in the external anal sphincter and toe flexors. Stimulation of the S1 root activated the hamstrings and gastrocnemius muscles and, to a lesser extent, the EAS. Once the root levels had been identified, the dorsal roots were stimulated with a pulse train (1 s, 50 Hz). Stimulus strength was, in most cases, below 2 mA. The S2 to the L2 roots were stimulated first. This was done in order to identify from which segments the most pathological reflex responses would be expected. Also, if stimulation of a root did not evoke any reflex response, this root was not divided into rootlets and considered for rhizotomy. Likewise, if the S2 root gave rise to EAS responses only and no toe flexion, the root was left intact. When a root had been divided into rootlets, all of these were stimulated before deciding which ones to cut or to spare. This was done to ensure that the rootlets with the most pathological reflex responses were cut. The number of rootlets cut was determined from the occurrence of pathological reflex responses in combination with preoperative assessments of spasticity interfering with motor function.

The number of sectioned rootlets was presented as the percentage of the total number of rootlets. However, since, in some cases, the S2 and/or L4 roots were left intact without being divided into rootlets, the median number of rootlets obtained from the other children was used for the calculation. For S2, this was six rootlets and, for L4, nine.

### Pre- and postoperative clinical examination and assessments

The families were questioned about the child's appetite and sleep, micturition and bowel habits, epilepsy, infections including urinary tract infections and other health problems preoperatively, at discharge from the habilitation centre, and at all postoperative controls. In addition, pain and sensory disturbances such as hypo- and hyperestesia were investigated. The scar after the SDR operation was inspected. The spine was clinically screened for scoliosis, hyperlordosis, kyphosis and pain/discomfort. Bladder scan or full urinary tract ultrasound examinations were performed to measure bladder emptying before and six months after SDR for all children operated 1997 and later. Weight and height were measured in a standardized manner preoperatively and at all follow-ups and plotted into Swedish growth charts [32,36].

At the pre- and postoperative follow-ups, deep tendon reflexes and clonus in the extremities were examined for each child by the same neuropaediatrician (JL or LW). The degree of deep tendon reflex response was rated in a 5-point scale, the National Institute of Neurological Disorders and Stroke (NINDS) scale [37]. Clonus was graded in a three point scale: no clonus, 1–6 beats, and ≥ 7 beats, i.e. inextinguishable clonus.

The muscle tone of the hip adductors, hamstrings and ankle plantar flexors was rated with the Ashworth scale, modified by Peacock, ranging from hypotonic (0) to parts rigid in flexion and extension (5) [7]. Passive range of motion (PROM) was measured for hip abduction with the knees extended, for popliteal angle and for ankle dorsiflexion with the knee extended and the foot inverted. The joint angles were measured at maximal range of movement with the use of a goniometer and standardised anatomical landmarks and methods as per the American Academy of Orthopedic Surgeons [38].

Spinal radiographs were performed preoperatively primarily to discover spinal anomalies that could interfere with the operation technique surgical procedure. Hip radiographs were taken in anterioposterior position preoperatively and were also performed at least at 5 years post SDR, migration percentage (MP) was calculated to identify hip subluxation.

### Outcome measures

The Gross Motor Function Measure (GMFM) is a measure of a child's gross motor function in a standardised observational way. It was designed to yield an index of gross motor function enabling changes in function to be evaluated after interventions or monitored over time for children with CP. The GMFM is a criterion-referenced measure based on normal gross motor developmental mile stones; all items are achievable by a five-year old child without any motor disability. GMFM has three scoring alternatives for total scores; GMFM-88 total, goal total score and GMFM-66. Also in GMFM-88 it is possible to obtain scores for five separate dimensions A. Lying and rolling; B. Sitting; C. Crawling and kneeling; D. Standing and E. Walking, running and jumping [39]. A goal total score was calculated for each child as the mean of the individually selected GMFM dimension scores. The dimensions were selected based on the child's functional status, age and areas of interests. The children were tested and videotaped by the same PTs (EN and ALJ) at all follow-ups. GMFM-88 total, goal total scores and dimension scores were calculated. However, one child was not tested in GMFM dimension A at the assessment preoperatively. Therefore, GMFM-88 total score was not calculated for this child. By using the Gross Motor Ability Estimator (GMAE), a GMFM-66 score was obtained for all children [39].

The Pediatric Evaluation of Disability Inventory (PEDI) is a generic standardised instrument used by the multidisciplinary team for evaluating functional performance, programme monitoring, documentation of functional development and clinical decision-making [40]. The target group is children aged 0.5–7.5 years. However it is also suitable for children older than 7.5 years if their functional ability is below that of non-disabled 7.5 year-olds. The PEDI contains 197 items in three dimensions; functional skills, caregiver's assistance and modifications/adaptive equipment used. Each dimension has three domains: self-care, mobility and social function. Normative scores are available to the age of 7.5 years; scaled scores can be used for all ages [40]. In this study, the PEDI was completed preoperatively and at follow-ups as a structured interview of parents by the same PT (EN) using the Swedish translation of PEDI [41]. We have chosen to present the results of scaled scores from the two dimensions Functional Skills and Caregiver's Assistance in the two domains Self-care and Mobility. PEDI was introduced in 1994, thus five of the children operated before 1994 have no PEDI data.

### Ethics

According to Swedish National Board of Health and Welfare, clinicians are obliged to secure the quality of care by performing and reporting results of clinical studies in everyday practise. Approval from an internal review board is not required for this type of research. All participants or caregivers gave their informed consent to participate in this follow-up. Participants and all data have been handled according to the Helsinki convention.

### Data analysis – statistics

Nonparametric statistics were used. Friedman's test was performed to explore change over time (preoperatively, 6, 12, 18 months, 3 and 5 years) for GMFM-88 total and goal score, GMFM-66 scores and PEDI Scaled scores for Functional skills and Caregiver Assistance in Self-care and Mobility. Wilcoxon's signed rank's test was used to determine more specifically at what time during follow-up statistically significant changes in function appeared (in the intervals pre- 6 months, pre- 12 months, pre-18 months, pre- 3 years and pre- 5 years postoperatively). To examine the relationship between the percentage of cut rootlets and GMFCS levels, Spearman correlation was performed. Significance levels were set to p ≤ 0.01 to correct for multiple comparisons. The Statistical Package of Social Sciences (SPSS 15.0) was used for calculations.

## Results

All 35 children attended all five follow-up appointments during the five years, but did not complete all items.

### Outcomes on body function and body structure

The mean proportion of transected rootlets was 40% (SD ± 5, range 25–53) (Table 2).

**Table 2:** The amount of transected lumbal (L) and sacral (S) rootlets (%).

**Root levels**	**GMFCS levels I–V (n = 35)**			**GMFCS levels I–II (n = 9)**			**GMFCS level III (n = 10)**			**GMFCS levels IV–V (n = 16)**		
	**Mean**	**SD**	**Range**	**Mean**	**SD**	**Range**	**Mean**	**SD**	**Range**	**Mean**	**SD**	**Range**
L2	49	13.0	(20-75)	40	11.3	(20-62.5)	51	14.6	(25-75)	52	10.9	(20-70)
L3	42	16.1	(0-75)	32	11.4	(14-50)	50	15.5	(22-75)	42	16.0	(0-66)
L4	26	15.7	(0-71)	20	15.7	(0-50)	28	20.5	(0-57)	28	11.4	(0-57)
L5	44	15.9	(0-63)	43	16.0	(0-56)	34	18.6	(0-61)	51	9.6	(23-62.5)
S1	57	12.6	(17-83)	59	10.8	(40-83)	54	14.7	(17-71)	58	12.3	(25-75)
S2	10	12.7	(0-60)	12	12.2	(0-33)	9	10.6	(0-33)	10	14.3	(0-60)
L2-S2	40	4.8	(26-53)	37	3.2	(30-43)	39	5.2	(26-46)	42	4.4	(33-53)

After SDR, the deep tendon reflexes decreased (p < 0.001) in the lower extremities. In most cases, they were completely extinguished. No further change occurred during the five years follow-up. The biceps reflexes in the upper extremities also decreased after the SDR (p < 0.01).

Preoperatively, 32 of the 35 children had ankle clonus. In 23, it was inextinguishable. Postoperatively, ankle clonus was present in only three children, who were among the four first operated. At that time, transection of rootlets in S2 was restricted because of fear of bladder dysfunction. When clonus was gone postoperatively, it did not reappear.

Muscle tone in hip adductors, hamstrings and ankle plantar flexors decreased (p < 0.001) between preoperative and six months postoperative follow-ups and remained reduced over the five years.

PROM increased during the whole 5 years period for hip abduction, popliteal angle and ankle dorsiflexion for the group as a whole (p > 0.001). The largest changes were detected at six months after SDR. Children in the GMFCS I–II sub-group showed statistically significant improvements for ankle dorsiflexors (p = 0.008) and children in GMFCS III increased hip abduction (p = 0.009). For children in GMFCS IV–V, statistically significant improvements were seen in hip abduction (p = 0.001) and popliteal angle (p = 0.004). Between 3 and 5 years postoperatively, there was a tendency for decreased popliteal angle for all children, especially for those in GMFCS III.

One child (GMFCS IV) had undergone orthopaedic surgery before SDR; a bilateral adductor tenotomy. During the first five postoperative years, 15 children (42%) had orthopaedic surgery in the lower extremities, as previously reported for the same cohort [42]. The surgical interventions addressed distal structures in 10 children, mainly subtalar arthrodesis and achilles tenotomy. Preoperatively 10 hips in seven children had MP > 33%. At five year follow-up, eight of the preoperatively ten hips with MP > 33% had improved, two had deteriorated of which one had been referred to hip surgery (rotational osteotomy) to prevent hip dislocation.

Increased lumbar lordosis was observed in four patients at the five years follow-up. Three patients (GMFCS II, III and IV) had spondylolisthesis according to the radiographs. One of these children had occasionally back pain while the other two had no back symptoms. Five children had developed scoliosis (Cobb angles 11–23°). None of the children had a brace or had undergone spinal surgery at the five-year follow-up.

### Functional outcomes

**Figure 1:**
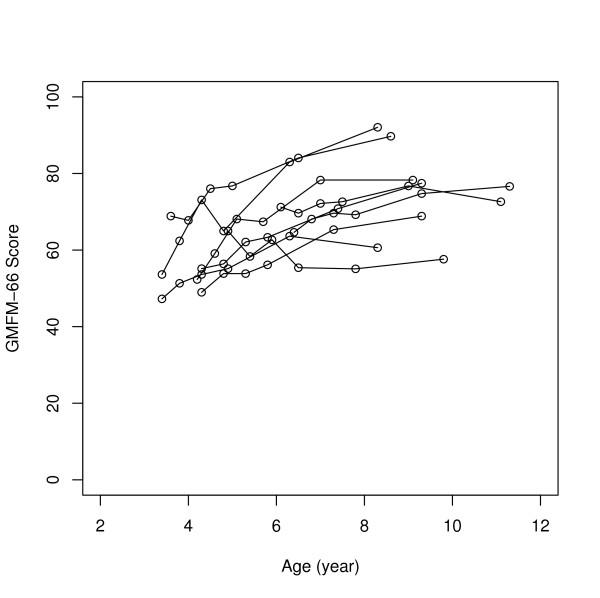
GMFM-66 development during five years postoperatively for individuals in the GMFCS levels I–II (n = 9).

**Figure 2:**
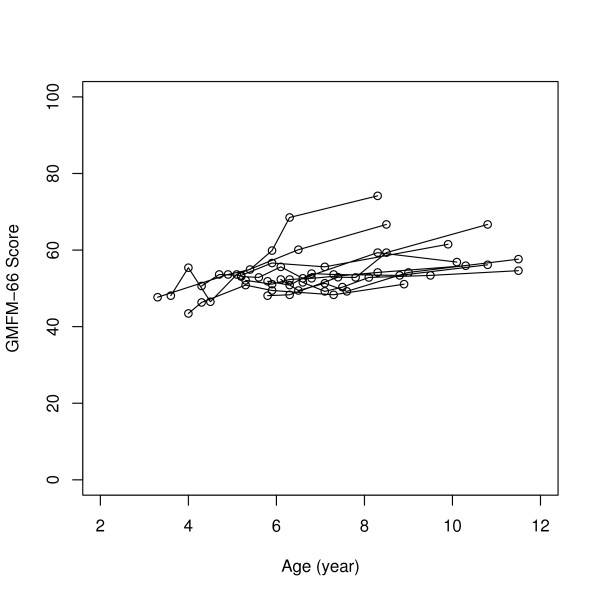
GMFM-66 development during five years postoperatively for individuals in the GMFCS level III (n = 10).

**Figure 3:**
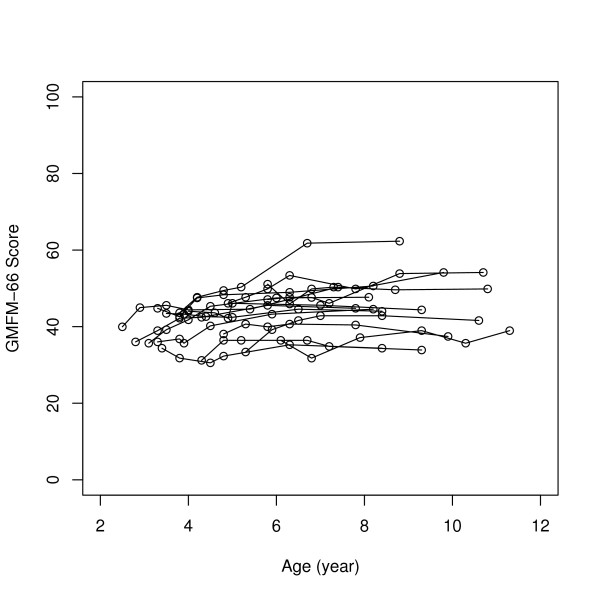
GMFM-66 development during five years postoperatively for individuals in the GMFCS levels IV–V (n = 16).

**Figure 4:**
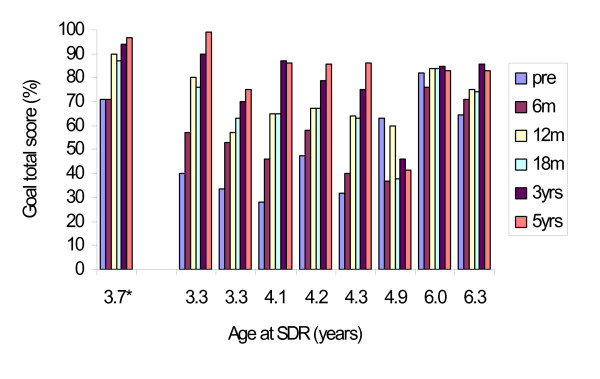
Individual GMFM goal total scores (%) for children in GMFCS-level I (n = 1*) and level II (n = 8), preoperatively, 6, 12, 18 months, 3 and 5 years postoperatively. The children are presented according to age at operation (beginning with the youngest child in the respective subgroup).

**Figure 5:**
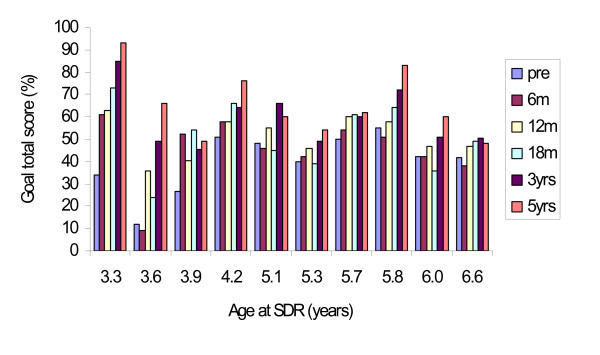
**Individual GMFM goal total scores (%) for children in GMFCS-level III (n = 10), preoperatively, 6, 12, 18 months, 3 and 5 years postoperatively.** The children are presented according to age at operation (beginning with the youngest child in the respective subgroup).

**Figure 6:**
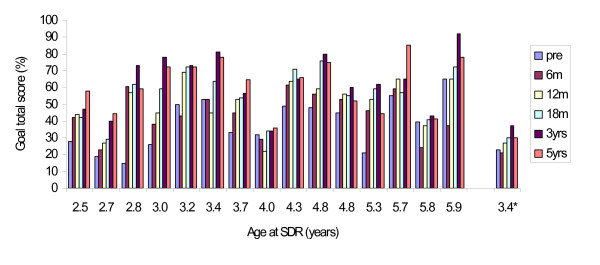
Individual GMFM goal total scores (%) for children in GMFCS-levels IV (n = 15) and V (n = 1*), preoperatively, 6, 12, 18 months, 3 and 5 years postoperatively. The children are presented according to age at operation (beginning with the youngest child).

The largest changes in scores between preoperative and 5 years were seen in the GMFCS levels I–II subgroup and the least changes in the GMFCS levels IV–V subgroup. For GMFM-66 for the separate GMFCS subgroups, significant changes were seen over the five years with Friedman's test (Table 3). Individual GMFM-66 development during the 5 years in the different GMFCS subgroups is illustrated in Figures 1, 2, 3, and individual GMFM-88 goal total scores in Figures 4, 5, 6. GMFM-66, GMFM-88 total and goal total scores showed statistically significant changes using the Wilcoxon's signed rank's test for the group as a whole at 1, 3 and 5 years postoperatively (Table 4).

**Table 3:** GMFM-66 baseline and mean change scores during 5 years.

**GMFCS level**	**Baseline**	**Preop-6 m**	**6–12 m**	**12–18 m**	**18 m-3 yrs**	**3–5 yrs**
	**Mean(SD) Range**	**Mean(SD) Range**	**Mean(SD) Range**	**Mean(SD) Range**	**Mean(SD) Range**	**Mean(SD) Range**
**I–II**	58.6 (8.9)	2.8 (4.9)	5.5 (4.4)	-1.2 (5.1)	7.5 (4.7)	2.1 (4.4)
	47.3–71.2	-5.5–10	0–13.7	-13.0–3.2	1.0–17.7	-4.1–9.1
						
**III**	49.9 (3.0)	1.0 (4.9)	1.8 (3.1)	1.3 (4.1)	2.2 (4.1)	3.7 (3.5)
	43.3–53.1	-6.5–9.8	-5.0–6.6	-3.0–8.5	-2.2–8.7	-2.5–7.4
						
**IV–V**	40.1 (5.8)	1.4 (3.1)	1.0 (2.0)	1.2 (2.0)	1.2 (3.7)	0.3 (2.5)
	31.2–51.1	-4.5–6.3	-2.8–4.8	-3.4–4.0	-2.6–11.9	-3.0–6.5
						
**I–V**	47.6 (9.8)	1.6 (4.0)	2.4 (3.5)	0.6 (3.5)	3.1 (4.8)	1.8 (3.6)
	31.2–71.2	-6.5–10.9	-5.0–13.7	-13.0–8.5	-2.6–17.7	-4.1–9.1

Mean baseline and change scores, standard deviation (SD) and range for GMFCS levels I–II (n = 9), III (n = 10), IV–V (n = 16) and GMFCS I–V (n = 35). P-values are obtained from Friedman's test for the GMFM-66 scores: pre-, 6, 12, 18 months, 3 and 5 years postoperatively.

**Table 4:** GMFM results.

GMFM score	Preop-12 m				Preop-3 yrs				Preop-5 yrs			
	Mean	SD	range	p-value	Mean	SD	range	p-value	Mean	SD	range	p-value
GMFM-66												
(n = 35)	3.5	4.8	-3.8-19.5	< 0.001	8.0	8.1	-9.9-29.4	< 0.001	9.5	9.7	-7.4-38.4	< 0.001
GMFM-88												
total (n = 34)*	10.9	10.3	-9.0-31.0	< 0.001	16.1	12.9	-4.0-52.0	< 0.001	21.2	17.9	-6.9-17.9	< 0.001
GMFM-88												
goal total (n = 35)	13.6	13.1	-10.0-42.2	< 0.001	23.6	17.4	-17.0-59.0	< 0.001	25.2	18.7	-21.3-59.0	< 0.001

Mean difference, standard deviation (SD) and range in GMFM-66, GMFM-88 total and goal total scores in children with spastic diplegia. P-values obtained with Wilcoxon's signed rank's test: pre- to12 months postoperatively, pre- to 3 years postoperatively, pre- to 5 years postoperatively. *One child was not tested in dimension A.

There were no statistically significant differences (Wilcoxon's signed rank's test) in GMFM-66 during the first 6 months, either for the whole group or for the GMFCS subgroups. For the whole group, statistically significant changes in GMFM-66 score were seen from 12 months postoperatively (pre- to 12 months postoperatively) and at all later follow-ups during the five years (pre- to 18 months, pre- to 3 years and pre- to 5 years postoperatively). For GMFCS levels I–II, changes were not statistically significant during follow-ups. Children in GMFCS III showed statistically significant changes after 3 and 5 years postoperatively and the children in GMFCS IV–V at 18 months, 3 and 5 years postoperatively.

The PEDI results for the whole group (n = 30) showed statistically significant changes (p < 0.001) with Friedman's test in the dimensions Functional Skills (Table 5) and Caregiver Assistance (Table 6) for the domains Self-care and Mobility. Statistically significant changes for scaled scores in both dimensions and domains were detected by the Wilcoxon's signed ranks test preoperatively to all postoperative follow-ups (pre- to 6 months, pre- to 12 months, pre- to 18 months, pre- to 3 years and pre- to 5 years postoperatively) for the whole group and for GMFCS IV–V. For children in GMFCS subgroups I–II (n = 8) and III (n = 7), there were no statistically significant improvements.

**Table 5:** PEDI scaled scores functional skills; self care and mobility.

	**Preop** **Mean (SD)**	**6 m** **Mean (SD)**	**12 m** **Mean (SD)**	**18 m** **Mean (SD)**	**3 yrs** **Mean (SD)**	**5 yrs** **Mean (SD)**	**p-value**
**Functional Skills, Self-care**							
GMFCS I–II	63.3 (9.1)	69.1 (10.4)	72.3 (10.4)	72.1 (7.0)	77.4 (7.3)	91.8 (10.1)	< 0.001
GMFCS III	59.6 (9.1)	64.0 (9.0)	65.2 (9.7)	67.2 (9.8)	70.7 (13.9)	75.9 (14.8)	NS
GMFCS IV–V	46.7 (6.2)	51.5 (6.6)	66.6 (6.8)	55.6 (6.8)	59.5 (8.1)	59.6 (8.3)	< 0.001
GMFCS I–V	54.2 (10.7)	59.1 (11.3)	61.4 (11.6)	62.7 (10.5)	66.9 (12.1)	72.0 (17.2)	< 0.001
**Functional Skills, Mobility**							
GMFCS I–II	63.9 (8.8)	71.8 (11.6)	75.1 (10.7)	79.7 (12.7)	82.8 (13.5)	86.0 (13.5)	< 0.001
GMFCS III	51.1 (9.1)	60.3 (9.0)	62.7 (5.8)	64.2 (9.8)	67.7 (6.2)	73.4 (11.8)	< 0.001
GMFCS IV–V	35.4 (7.6)	42.6 (8.3)	46.9 (8.1)	48.4 (8.1)	52.8 (8.5)	52.3 (14.3)	< 0.001
GMFCS I–V	46.7 (14.7)	54.6 (15.7)	58.1 (14.7)	60.5 (16.5)	64.2 (15.9)	66.2 (19.8)	< 0.001

Mean value, Standard deviation (SD) for all assessments in GMFCS I–II (n = 8), GMFCS III (n = 7), GMFCS IV–V (n = 15) and the group as a whole GMFCS I–V (n = 30). P-values obtained with Friedman's test: pre- 6, 12, 18 months, 3 and 5 years postoperatively.

**Table 6:** PEDI scaled scores caregiver assistance; self care and mobility.

	**Preop** **Mean (SD)**	**6 m** **Mean (SD)**	**12 m** **Mean (SD)**	**18 m** **Mean (SD)**	**3 yrs** **Mean (SD)**	**5 yrs** **Mean (SD)**	**p-value**
**Caregiver Assistance, Self-care**							
GMFCS I–II	58.7 (12.0)	61.9 (14.3)	62.5 (12.5)	67.3 (10.5)	73.2 (8.6)	88.8 (13.5)	< 0.001
GMFCS III	56.1 (10.9)	61.7 (10.1)	66.1 (16.7)	65.2 (8.2)	68.5 (14.6)	72.2 (15.6)	NS
GMFCS IV–V	34.9 (10.1)	42.0 (9.9)	45.0 (11.0)	47.7 (11.8)	52.3 (12.8)	53.3 (14.4)	< 0.001
GMFCS I–V	46.2 (15.5)	51.9 (14.8)	54.6 (15.8)	57.0 (14.1)	61.7 (15.3)	67.2 (20.7)	< 0.001
**Caregiver Assistance, Mobility**							
GMFCS I–II	62.0 (12.8)	70.4 (11.1)	75.9 (12.2)	79.3 (13.8)	89.3 (15.3)	93.1 (10.6)	< 0.001
GMFCS III	51.1 (9.1)	60.3 (8.9)	62.7 (5.8)	64.3 (9.8)	67.7 (6.2)	73.4 (11.8)	0.01
GMFCS IV–V	34.9 (10.1)	42.0 (9.9)	45.0 (11.0)	47.7 (11.8)	52.3 (12.8)	53.3 (14.4)	< 0.001
GMFCS I–V	43.4 (21.4)	52.3 (18.9)	56.7 (17.5)	60.6 (18.5)	67.8 (20.1)	71.7 (19.5)	< 0.001

Mean, Standard deviation (SD) for all assessments in GMFCS I–II (n = 8), GMFCS III (n = 7), GMFCS IV–V (n = 15) and the group as a whole (GMFCS I–V (n = 30). P-values obtained with Friedman's test: pre- 6, 12, 18 months, 3 and 5 years postoperatively.

### Postoperative medical observations

No major complications occurred peri- or postoperatively. In one child, laryngospasm occurred during the anaesthesia. One child had pneumonia, two obstructive bronchitis and three urinary tract infection during the first postoperative week. Four children had cerebral spinal fluid leakage for a few days. Four children had insufficient postoperative pain control, while five had pain breakthrough which was easily treated. Four children had transient problems with micturition after removal of the indwelling catheter. Dysesthesia was common, mostly of 2–3 weeks duration. Muscle spasms in thighs and back occurred in five children and were treated with diazepam with good effect. Twelve children had constipation problems the first weeks. All children could be discharged from the hospital after 3 1/2 weeks according to the preoperative plan.

Twenty-seven of the 35 children had no problems at all with micturition, bowel habits, sleep or pain during the period three months to five years postoperatively. Occasional urinary infection or incontinence, constipation or sleep problems were present in seven children during the follow-up period; while one had intermittent back pain five years post SDR. No new urinary tract problems, including incontinence, were present after the SDR-operation. Many children had problems with constipation preoperatively, in five the problems disappeared postoperatively. Nine still needed medication for constipation five years postoperatively.

Five years postoperatively, the two children with preoperative overweight were still obese and two others had become overweight. One child had acquired underweight. Three of the 11, children who were underweight before the operation, were still underweight.

Sensory problems ascribed to the surgical procedure were dys- or hyperaesthesia, which had disappeared, in all but three children, six months postoperatively. One child had recurrent hyperaesthesia and flexor spasms during febrile infections, the sensory problems were successfully treated with anti-epileptic medication. One child had recurrent wounds on the lateral aspect of the left fifth toe with clinical signs of hyposensitivity. One child had persistent problems with keloid formation in the scar and itching five years postoperatively.

## Discussion

Our results support that SDR is a safe, effective, durable spasticity-reducing treatment for children with spastic diplegia in all GMFCS levels. SDR reduces spasticity immediately and permanently. The mean proportion of transected rootlets in our study was in accordance with the meta-analysis study made by McLaughlin et al. [21]. In combination with physiotherapy, it leads to a beneficial outcome of quantitative gross motor function, performance of functional skills and activities, as well as increased independence in self-care and mobility at least up to 5 years postoperatively. These results are in accordance with recent studies [18,19,26,43]. No serious complications due to the surgical intervention occurred. The instrument constructed to separate the rootlets from each other made the procedure of stimulation, selection and transection of the rootlets safe.

This is a prospective long-term follow-up study of a consecutive, complete series of SDR-operated children. When introducing SDR in 1993 we did not have the possibility to create a comparison group. Instead we chose regular and thorough follow-ups and a long-term perspective on development of different aspects of function, a practice-based evidence approach to evaluate the outcome [44,45].

The appropriate selection criteria are crucial and it is suggested that preoperative diagnosis is the strongest predictor of outcome after SDR [8]. The selection criteria formulated by Peacock have not been changed during the study period [7]. However we did not exclude children with cognitive disabilities if they had a drive to move and interact in playful treatment situations. Children had to have spasticity which seriously interfered with and inhibited their further motor function development and daily care. As described by Steinbok [43]; it is critical to ascertain what the expectations and hopes of the parents or caregivers are and to ensure that they are realistic. One must discuss what happens when part of the spasticity is relieved and in what situations the child might have had use of preserved spasticity i.e. weight-bearing, standing, mobility, transfers and self-care. This is important to consider for both the children with and without ability or potential to walk.

The selected children were young and had not yet developed manifest contractures. The importance of an early SDR operation has been stressed by orthopaedic surgeons [24]. Our experience is that children at an age of 2–5 years are easy to motivate and engage in playful physiotherapy sessions. This is a time, before school starts, when it is natural and easy to focus on activities in locomotion and mobility. Fifteen (42%) of the children in this cohort had undergone minor orthopaedic surgery (mainly to address planovalgus) at 5 year follow-up [42]. There are variability of rates of orthopaedic surgery depending on age, severity and different indications for ortopaedic surgery between centers. Subramanian et al. reported the need for additional orthopaedic surgery after SDR in 65% [23] and Caroll et al. in 45% [46]. O'Brien et al. found lower rates of orthopaedic surgery for children undergoing SDR in the ages 2–5 years (34%) than for children undergoing SDR in the ages 6–14 years (70%) [24].

It has also been discussed if SDR has a positive or a negative influence on the development of spinal deformities. Golan et al. [47] found that younger age at surgery was associated with a lower rate of hyperlordosis/spinal deformities. They also reported mild scoliosis in 45% and 19% spondylolisthesis in a sample of 98 children undergoing SDR with follow-up radiographs at a mean of 5.8 years after SDR. Farmer et al. [26] also showed that children who had undergone SDR before five years of age had less lumbar lordosis than the children operated at an older age. In our study, two of the four children with increased lumbar lordosis at follow-up were below the age of 5 years (4.0 and 4.1) when undergoing SDR. Five years postoperatively, none of the children in our series had needed any treatment for spinal deformity. These results are in accordance with the study by Golan et al. in which no child experienced clinically significant deficits [47].

Long lasting benefits in PROM were seen for the group as a whole in hip abduction and ankle dorsiflexors. However, between three and five years postoperatively, there was a tendency for a decreased range of motion in the popliteal angle especially for GMFCS III. This is an important observation for postoperative treatment planning.

The overall positive results of PROM in this group of children might be due to multiple factors including low age at SDR, recommendations to children in all GMFCS levels to use a standing shell for at least 1–2 hours per day, regular follow-ups with goal directed physiotherapy interventions and use of orthoses. Probably all additional treatments above have gained PROM development and hip status. The isolated role of SDR is not examined by this study design. Severe contractures and hip subluxation are rare in our sample, but we cannot state a lower frequency of contractures than without SDR, due to lack of information about contracture development in other children with spastic diplegia.

Children with cerebral palsy (CP) are generally short and many, especially non-ambulating children are severely underweight and stunted [48]. There are no longitudinal growth data on optimally fed children with CP and therefore, we chose to use the reference population growth curves. The arbitrarily chosen BMI and weight cut-off for over- and under-weight in this study were wider than the WHO definitions for typically developing children. In the present study, many of the children who were preoperatively underweight normalized their weight, probably as a consequence of the loss of the preoperative spasticity induced energy consumption. The rate of weight gain needs to be brought to attention and dealt with as the children are getting older, taller and heavier. It is of great importance to avoid contractures and weakness at the knees to prevent crouch position due to lever-arm dysfunction, too much sitting and lack of activity.

The impact of growth, additional treatment, such as amount of physiotherapy, and orthopaedic surgery are confounding factors in the long-term follow-up after SDR. We regard the different intervention options as complementary rather than exclusive and inform our patients and families accordingly. Thus a continued multidisciplinary interaction and follow-up is necessary. Regular and systematic follow-up is of great importance especially during growth spurts.

We have chosen to use multiple evaluative and standardized classifications and measures on body function and structure, and on activities and participation as an integral part of our clinical routines. The GMFM is the most commonly used instrument for detecting change in gross motor function in children undergoing SDR. However, it has to be kept in mind that the GMFM is constructed to measure quantitative aspects i.e. how much children can do, not the quality nor the performance of movements and skills. Our impressions are that children are gaining quality and flexibility of movements: ease, smoothness and improved sitting which affects arm and hand function. These aspects of function are not possible to score using the GMFM.

Prior to SDR, the children had some positive functional effect of their spasticity. The immediate spasticity reduction after SDR facilitates flexibility and variability in lying, sitting, crawling, and kneeling positions. However, weight-bearing in standing, alignment and postural control in transfers and locomotion requires voluntary strength which takes longer time to achieve postoperatively. As expected, there were no statistically significant improvements during the first 6 months postoperatively. It takes time to develop strength and to gain new functional skills and use them in complex activities. Strength was not measured pre- and postoperatively due to lack of valid standardized measures reliable enough for assessments in early pre-school years. Instead strength was estimated in complex functional skills and activities.

The development in gross motor function was improved over the five years. Thus if functional outcomes should be detected, it is important to follow the children at least five years postoperatively. When comparing results from different follow-up studies, it is important to consider differences in sample characteristics and GMFCS levels as well as the time interval and length and frequencies of drop-outs during the follow-ups.

Most follow-up studies present final outcomes with GMFM-88 one to two years after SDR [9,10,12,16,20]. To our knowledge, GMFM-66 is rarely used as the main outcome measure. However, in the study by McLaughlin et al. [21] both GMFM-66 and GMFM-88 were used in the meta-analysis at 9–12 months follow-up. The mean GMFM-66 change of scores for the pooled data was 2.7 and for GMFM-88 total score 4.5. It was concluded that SDR and physiotherapy were effective in reducing spasticity and had small positive effect on gross motor function. In our study, the mean GMFM-66 change score at the 12 month postoperative follow-up was 3.5 and for the GMFM-88 total score 10.9. Mittal et al. [18] presented GMFM-88 mean change scores at 1, 3 and 5 year follow-up of 10.1, 19.9 and 34.4 respectively. The corresponding mean changes in our study were 10.9, 19.1 and 21.2, respectively, which are similar during the first three years but lower at five year follow-up. The reason for this might be that Mittal et al. did not include non-ambulatory children (GMFCS IV–V).

Our results show great variability in GMFM scores between different GMFCS levels, which has also been reported earlier for GMFM-88 [49] and GMFM-66 [50]. Therefore, our results have been presented for the group as a whole as well as for the three subgroups. The sample sizes in the subgroups were small and, therefore, the results might be influenced by type II-error. Nevertheless, in this study, the GMFM-66 results showed statistically significant increase in gross motor function capacity for the separate subgroups over the 5 years.

To identify the goals of each individual child, we have found GMFM-88 goal total score and separate GMFM-88 dimension scores useful in planning and evaluating goal-directed interventions. All patients but one improved their goal scores. An unexpected decline in motor function occurred in a child with preoperative GMFCS level II (SDR at 4.9 years, Figure 4). The child was born at term. The pregnancy, delivery and the first two years of life were uncomplicated. A cranial tomography was performed due to deteriorated gait at three years of age and was found to be normal. Between this age and the SDR operation at five years of age, no further deterioration was noted. During the first postoperative year, motor function improved, but later leveled off and declined. Two immobilization periods due to fractures and orthopaedic surgery probably contributed, but a progressive disorder cannot be ruled out.

Fifteen children in our study had preoperative gross motor function classified as GMFCS level IV. Their mean age at SDR operation was 4.1 years, (SD 1.7). It is known that children with GMFCS IV–V are expected to reach 90% of their potential GMFM-66 scores at an age of 3.5 years (range 3.2–4.0) [50]. Many of our severely disabled children continued to gain in motor function even after the age of 3.5 years (Figure 3 and 6). Two children in GMFCS level IV, who had minor improvements over the five years, had cognitive disabilities including autism with difficulties to participate in physiotherapeutic training and test situations (SDR at 4.0 and 5.8 years). In contrast, one child (SDR at 3.7 years) with severe mental retardation/learning disability had a great improvement after SDR (Figure 6).

Twice as many boys than girls were selected for SDR, mainly reflecting gender distribution of CP spastic diplegia in our area (prevalence 1.3/1000 boys and 0.7/1000 girls) [51]. The best GMFM outcome was found in nine of the 24 boys and four of the eleven girls, i.e. the proportion is the same for boys and girls. In our study group, with a comparatively narrow age range, age at operation did not correlate to GMFM outcome.

PEDI was used to broaden the perspective of function and highlight performance; assessing what the individual child actually does in meaningful daily activities in contrast to capability; what they can do in specific test situations which were measured with GMFM. Traditionally, improved walking has been a main goal for children undergoing SDR. However, in the present study, the children with more functional limitations (GMFCS IV–V) never had walking as a goal. It has been shown that there is a great variability in mobility in children with CP, even within the same GMFCS level, due to contextual, environmental and personal factors [52].

The advantages of using PEDI are to explore the parent's view of the most common performance of the child in the home environment and the amount of caregiver assistance. In addition, it is a valuable tool in the rehabilitation process to define functionally realistic goals and modifications needed for independence in activities in daily life. In the present study, the largest functional improvements appeared in the early postoperative follow-up. The improvements continued during the five years, which is in accordance with Mittal et al. [19]. We concluded that both GMFM and PEDI are clinically useful for these patients and provide complementary information on functional outcome in a long-term follow-up.

## Conclusion

SDR is a safe and effective method of reducing spasticity permanently without major negative side effects. In combination with physiotherapy, it provides lasting functional benefits in a group of carefully selected and systematically monitored young children with spastic CP. Improvements over time were seen during the five year follow-up period. As the goal of treatment is to improve activities and participation, a long-term, multidisciplinary follow-up of all the children is necessary. This cohort is continuously and systematically monitored by the same team to answer crucial questions about their function as adolescents and young adults.

## Abbreviations

BMI: Body Mass Index; CP: Cerebral Palsy; GMFCS: Gross Motor Function Classification System; GMFM: Gross Motor Function Measure; MP: Migration Percentage; PEDI: Pediatric Evaluation of Disability Inventory; PROM: Passive Range of Motion; SDR: Selective Dorsal Rhizotomy.

## Competing interests

The authors have no financial interest in this study, which was performed as part of a clinical follow-up programme.

## Authors' contributions

EN, ALJ, JL and LW planned administrated assessments and measurements and analyzed the results of all follow-up data. EN, ALJ and LW wrote and revised the manuscript with active assistance of JL, GA and LGS.

## Pre-publication history

The pre-publication history for this paper can be accessed here:


